# Auditory Evoked Bursts in Mouse Visual Cortex during Isoflurane Anesthesia

**DOI:** 10.1371/journal.pone.0049855

**Published:** 2012-11-21

**Authors:** Rüdiger Land, Gerhard Engler, Andrej Kral, Andreas K. Engel

**Affiliations:** 1 Department of Neurophysiology and Pathophysiology, University Medical Center Hamburg-Eppendorf, Hamburg, Germany; 2 Department of Experimental Otology, Institute of Audioneurotechnology, Hannover Medical School, Hannover, Germany; University of Alberta, Canada

## Abstract

General anesthesia is not a uniform state of the brain. Ongoing activity differs between light and deep anesthesia and cortical response properties are modulated in dependence of anesthetic dosage. We investigated how anesthesia level affects cross-modal interactions in primary sensory cortex. To examine this, we continuously measured the effects of visual and auditory stimulation during increasing and decreasing isoflurane level in the mouse visual cortex and the subiculum (from baseline at 0.7 to 2.5 vol % and reverse). Auditory evoked burst activity occurred in visual cortex after a transition during increase of anesthesia level. At the same time, auditory and visual evoked bursts occurred in the subiculum, even though the subiculum was unresponsive to both stimuli previous to the transition. This altered sensory excitability was linked to the presence of burst suppression activity in cortex, and to a regular slow burst suppression rhythm (∼0.2 Hz) in the subiculum. The effect disappeared during return to light anesthesia. The results show that pseudo-heteromodal sensory burst responses can appear in brain structures as an effect of an anesthesia induced state change.

## Introduction

General anesthetics modulate cortical network properties in a dosage dependent manner. Changes in ongoing activity during increase of anesthesia level are paralleled by changes in sensory evoked responses. Anesthetic dosage modulates receptive field properties [1], [2] as well as response strength and response variability [3], [4]. Anesthetics affect ongoing neuronal activity, with an anesthesia dependent increase in correlation between cortical neurons, and a related increase in coherence of ongoing activity [5]–[7]. Increasing levels of anesthesia eventually produce burst-suppression of cortical activity, a pattern of alternating high amplitude bursts and periods of suppressed activity (silent periods) signifying deep anesthesia [8]–[10]. Deep anesthesia, however, does not simply produce a loss of brain responsiveness to sensory stimuli, but induces global changes in cortical reactivity [11]–[16].

Little is known about the evolution of cortical response properties between light and deep anesthesia. Cortical network properties clearly differ between light and deep anesthesia, but it remains unclear when these properties change during deepening of anesthesia and whether there is a gradual transition or a discontinuous change in cortical excitability. To address these questions, we performed simultaneous multisite recordings in primary visual cortex (V1) and the subiculum of mice during continuous change of anesthesia level. Although we were mainly interested in anesthesia effects on visual cortex, the use of a single shank multisite electrode allowed simultaneous sampling of activity in the subiculum, and thus we could compare the anesthesia effects in visual cortex to a not directly anatomically connected non-sensory structure. We continuously measured the effects of visual and auditory stimuli on activity during the evolution from light to deep anesthesia. The results of this study show a distinct state change between light and deep anesthesia. In light anesthesia, primary visual cortex did not respond to auditory stimuli. However, at some point during the evolution into deep anesthesia auditory stimulation began to induce activity in V1, and both visual and auditory burst responses could be evoked in the subiculum. This state of increased sensory excitability was associated with the presence of a stable slow rhythm (∼0.2 Hz) in ongoing subicular activity. The heteromodal responses disappeared as soon as isoflurane concentration dropped below a critical level and was not present during light anesthesia.

## Materials and Methods

### Surgical Preparation

15 female C57/Bl6 mice, weight 20–25g, were used in this study. Anesthesia was induced by intraperitoneal injection of Ketamine (100mg/kg) and Xylazine (4mg/kg). Tracheotomy was performed and mice were mechanically ventilated. Anesthesia was maintained for the rest of the experiment with isoflurane vaporized in a 3∶1 mixture of O_2_ and N_2_0. Mice were placed in a custom built head holder and the head was fixated for surgery by means of a mouth/nose clamp and two non-traumatic ear bars. During auditory stimulation the ear bars were released. Electrocardiogram (ECG), heart rate, body core temperature and end-expiratory CO_2_ concentration was continuously monitored. End-expiratory CO_2_ concentration was monitored with an adapted carbon dioxide monitor (Capstar-100, CWE Inc., USA) throughout the whole experiment and kept below 4%. During preparation and surgery pedal (paw pinch) withdrawal reflexes and presence of whisker movements were monitored, and anesthetic dose was adjusted as required. The right visual cortex (A17) was exposed by craniotomy at -3.8mm posterior to bregma and 2.7 mm lateral to lambda [17]. The dura was left intact, and mineral oil was applied to the cortical surface to prevent dehydration. The reference electrode was a silver-plated wire inserted onto the surface of the frontal cortex under the skull, through a second small craniotomy. It was fixed in place with bone wax and tissue adhesive (Histoacryl, Braun-Aesculap, Tuttlingen, Germany). All procedures were approved by the Hamburg State Authority for Health and Consumer Protection (BGV Hamburg, Germany).

### Data Acquisition

Simultaneous recordings were performed from V1 and the subiculum using one-shank 16-channel multisite probes (NeuroNexus Technologies, AnnArbor, MI, USA). Probe sites were separated vertically by a distance of 100 µm. Electrode site area was 177 µm^2^ with impedances of ∼1MΩ at 1 kHz. The multiprobe was inserted orthogonally to the cortical surface and was advanced into the brain with a mechanical micromanipulator until the topmost recording site was located at the surface of the cortex. In this manner, the 16 recordings sites spanned the depth of V1 and locations in the subiculum to a depth of ∼1500 µm from the cortical surface. Electrode signals were recorded and digitized using an Alpha Omega recording system (Alpha Omega Engineering, Nazareth, Israel). The electrode signals were split into a low pass filtered (600 Hz) signal sampled at 3125 Hz and a band-pass filtered at 300–5000 Hz with sampling at a sampling rate of 25 kHz.

### Electrode Position in V1 and Subiculum

**Figure 1:**
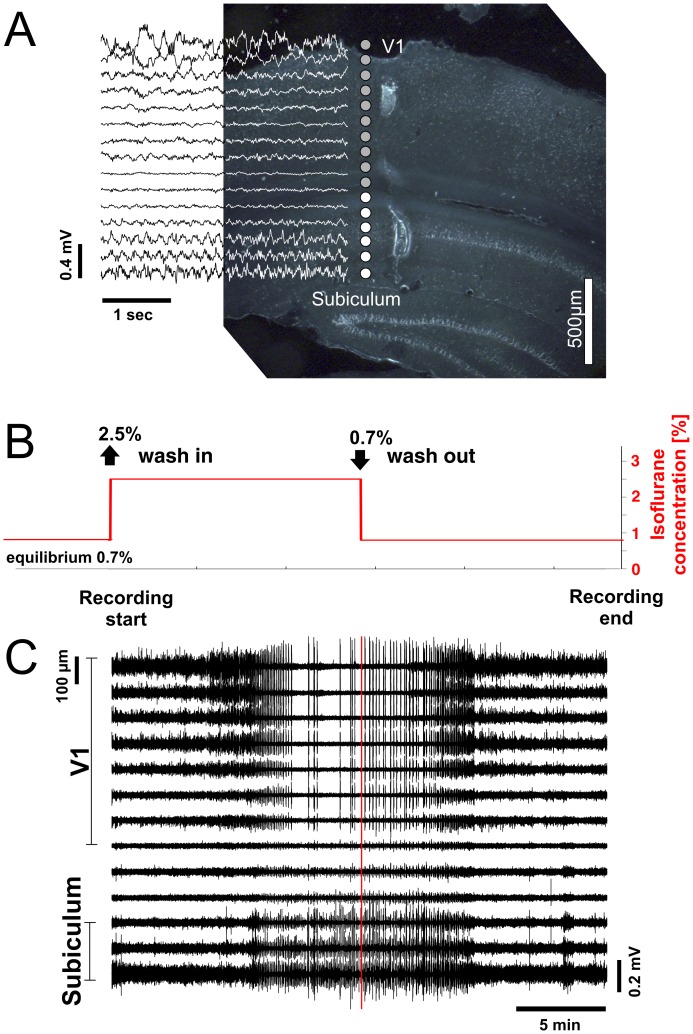
Ongoing LFP activity in V1 and subiculum during continuous change of anesthesia level. (A) Reconstruction of electrode site location. Upper electrode sites are located in V1 (gray dots) and lower sites in subiculum (white dots). Traces show LFPs during light anesthesia at 0.7% isoflurane baseline equilibrium. Note that LFPs shown are bipolar derivations (see methods) between adjacent electrode sites, resulting in only fifteen signal traces displayed between the sixteen electrodes sites (dots). (B) Experimental protocol. From baseline equilibrium at 0.7%, isoflurane concentration (red line) was increased in one step to 2.5% and subsequent changes in activity were recorded (wash-in). After ∼15min isoflurane concentration was decreased to 0.7%, and again changes of neuronal activity were recorded for ∼ 15min (wash-out). (C) Depth profile of LFP activity during one full cycle of wash-in and wash-out of isoflurane. LFP represent bipolar derivation between neighboring electrodes. Time scales in (B) and (C) are matched. Red line in (C) marks start of wash-out. Distance between electrode sites was 100 µm.

Electrodes were positioned with the topmost site at the surface of the cortex. To assign electrode positions to V1 and subiculum we used the following criteria. The obtained activity profiles varied with depth in a regular manner. Strong unit activity usually appeared at electrode site 4 and 5. Electrode sites positioned in or near white matter at a depth ∼1000 µm (Figure 1A) showed a prominent drop in amplitudes of local field potentials (LFPs) and no spiking activity. This drop was taken as an indicator of the border between V1 and subiculum. To verify recording positions, electrolytic lesions (15µA for 15s) were performed and subsequently identified histologically in three of the animals. Injecting current may damage the electrodes, therefore we did not use this procedure in all of the experiments. Additionally, the silicon probes left tracks that were discernible in fixed Nissl-stained sections. In all animals, the electrode penetrated through the entire depth of the cortex into the white matter and below into the subiculum. Using the histological and physiological evidence we observed electrodes 2–7 to be clearly located in V1 and electrodes 12–15 in the subiculum, which were then included in the subsequent analyses of signals from the two structures. The non-assigned electrodes were considered ambiguous, and excluded from the analysis.

### Modulation of Isoflurane Level and Recording Protocol

After achieving an equilibrium anesthetic state at 0.7% vol isoflurane concentration, recording was started and the anesthesia concentration was increased in one step to 2.5% vol isoflurane. Wash-in of anesthetics was continued until long periods of isoelectric LFP in recordings appeared and indicated pronounced suppression activity. Then, isoflurane concentration was switched back to 0.7% and recording continued until the initial state was reached again. In all animals tested, both the wash-in and wash-out took about 15 min each. This cycle was repeated twice for recording of changes in ongoing activity and of changes in stimulus-related activity, respectively. The order of the respective two cycles for ongoing and stimulation were randomly assigned before the experiment.

### Visual and Auditory Stimulation

In the stimulus presenting condition, auditory and visual stimuli of 1s duration were presented in alternation continuously during the whole cycle of isoflurane wash-in and wash-out, with interstimulus intervals between the stimuli of 5s. Visual stimuli were generated using Matlab (Mathworks, Natick, MA, USA) with the Psychophysics Toolbox [18], [19] on a Mac Pro (Apple, USA). As visual stimuli, full field flash stimuli with 1s duration were presented to the contralateral eye using a cathode ray tube (CRT) monitor (Iiyama Vision Master Pro 451) with refresh rate set to 100 Hz, and luminance values ranging between 0.2 to 98cd/m^2^ (measured with Luminance Meter LS-100, Konica Minolta). Auditory stimuli were 1.3 kHz pulse trains presented at ∼70dB LAeq (measured with Brüel&Kjær hand-held Analyzer Type 2250-L, Denmark) via a speaker (DT48, Beyerdynamic GmbH & Co KG, Germany) positioned at a distance of 50cm from the animal’s head (free-field configuration).

### Signal Post-Processing

Data were exported to Matlab (The MathWorks, Natick, MA) for further processing. Three types of signals were derived from the raw data. LFPs were obtained by low-pass filtering with a 4^th^ order Butterworth filter with a cutoff frequency of 100 Hz. After filtering, LFPs were resampled at 500 Hz. In order to eliminate far-field effects, the LFP signals were further processed by applying a bipolar derivation between neighboring sites, which yields the potential difference between adjacent probe contacts (see also [20]). Neighboring channels were subtracted, resulting in a bipolar derived signal in a total of 15 channels. After bipolar derivation simultaneous oscillations of identical voltage at both probe contacts will be cancelled out. Consequently, the resulting LFP signals revealed clear differences in activity patterns between visual cortex and the subiculum (see Figure 1A) previously covered by far-field effects. Further, during deep anesthesia with presence of LFP burst suppression pattern, the high amplitude LFP bursts correlated always with bursts of mulitunit activity, and no multiunit activity was present during the suppression periods. In conclusion, the resulting LFP activity reflects local activity between the respective electrode sites. To extract the multiunit activity (MUA), the 25 kHz signal was first rectified and then low-pass filtered with a 4th order Butterworth filter with a cutoff frequency 100 Hz. Then, the resulting signal was resampled at a sampling rate of 500 Hz. The MUA obtained in this way is thought to represent a continuous weighted average of the extracellular spikes of all neurons within a sphere around the tip of the electrode. To extract spike times from the 25 kHz signal, the threshold for spike detection was set at 3.5 SDs. A spike was recognized as such only if the last spike occurred >1 ms earlier.

### Definition of Cortical Suppression Ratio

The cortical suppression ratio (CSR) was determined by calculating the ratio of time within a 30 second interval, in which the rectified LFP signal was below a fixed threshold. The threshold was chosen as the median of the rectified signal of one full recording cycle of wash-in and wash-out (26µV mean ±15µV SD, varying with the depth of the electrode site, with larger values of LFP median on the surface and in the subiculum). Then the CSR was determined for shifted intervals along the full recording, with a sliding window size of 30s and a step size of 1s. This resulted in the time course of the index over the whole recording length. This procedure was performed for LFP signals from the upper cortical electrodes (3–6) in V1, and the individual results from the four sites were then averaged, resulting in the CSR. A value of 1 corresponds to full suppression of cortical LFP activity, whereas index value 0 would theoretically correspond to full activation, with the signal being above threshold during the whole window length (DC shift).

**Figure 2:**
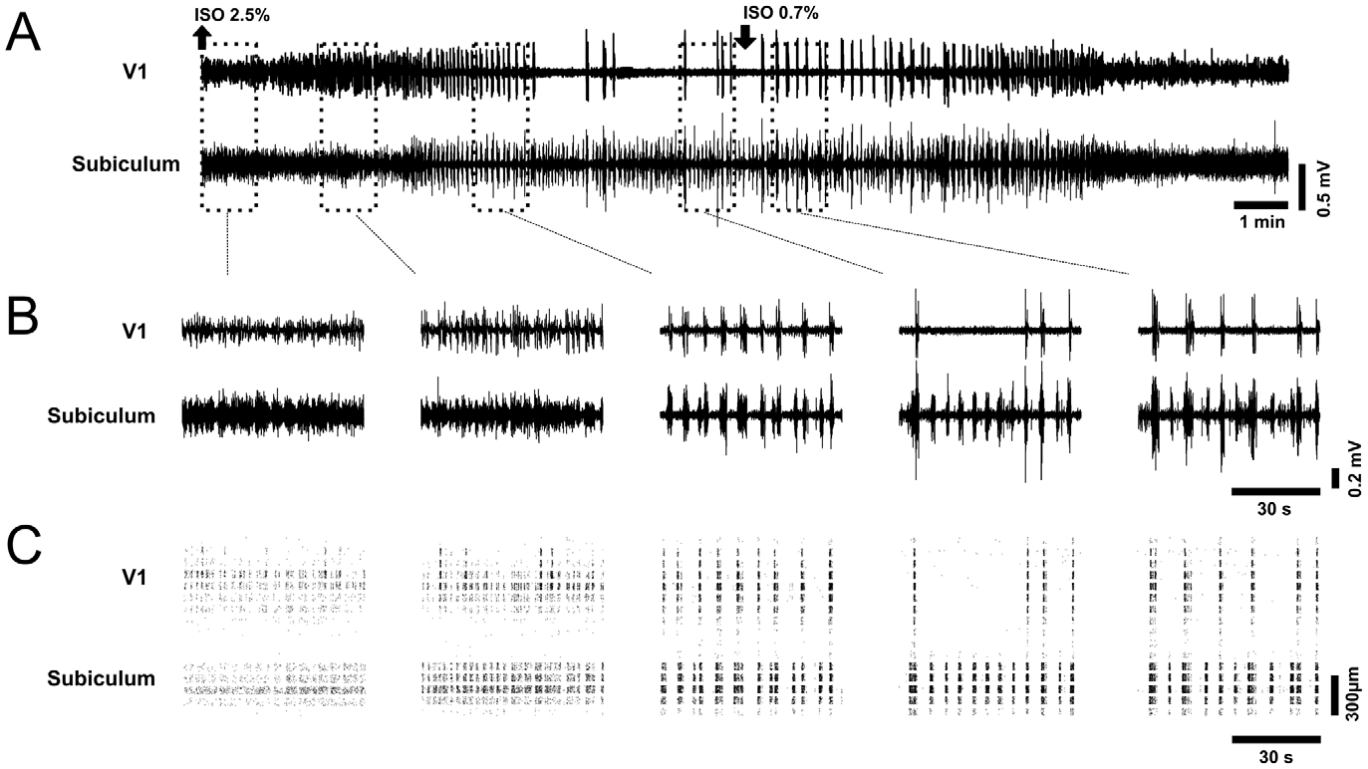
Differences in ongoing LFP activity in V1 and subiculum. (A) Comparison of LFP activity in V1 and subiculum during full cycle of wash-in and wash-out of isoflurane. Shown are LFPs from electrode site 3 and electrode site 12 from 1C. Black arrows depict the onset of wash-in and wash-out, respectively. (B) Expanded one minute segments of LFP activity during different stages of anesthesia (red dotted rectangles in A). (C) Corresponding depth profile of multiunit activity of time segments shown in (B). Note that bursts of multiunit activity always correlate with bursts in LFP activity in the respective regions. V1 and subiculum are separated by electrode sites with nearly absent multiunit activity. Each band shows raster plots of multiunits of one electrode site. Points in a raster band have been spread in their vertical position in the range of the band to increase visibility (see methods).

### Definition of Suppression Levels

In order to pool sensory responses during similar CSR we defined five suppression levels (1 through 5). The suppression level of activity was defined with respect to the CSR values. CSR <0.4 corresponds to level one, CSR between 0.4 and 0.5 to level two, 0.5 and 0.6 to level three, 0.6 and 0.7 to level four and level five corresponding to CSRs >0.7. The classification into five suppression levels allowed to average the stimulation trials obtained in each level, and thus to better visualize the effect of anesthesia level on averaged visual and auditory responses in V1 and the subiculum.

**Figure 3:**
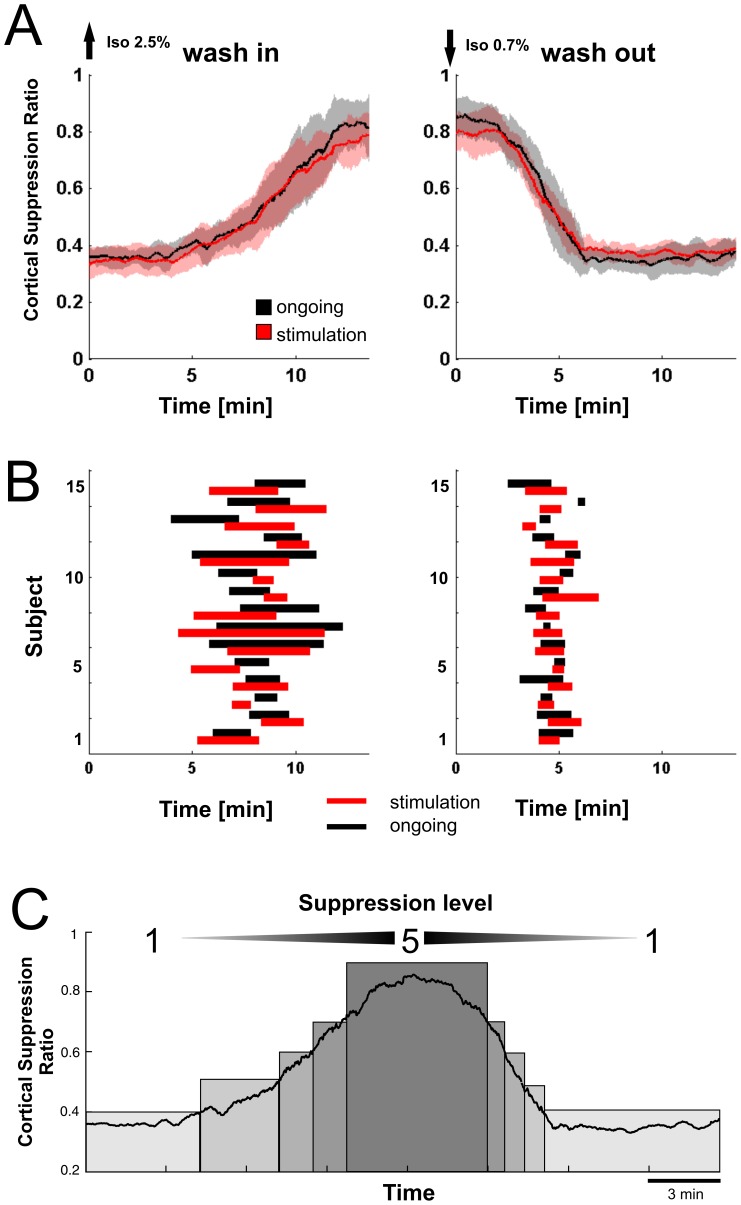
Cortical suppression ratio (CSR) and definition of suppression levels. (A) Changes in cortical suppression ratio during wash-in (left panel) and wash-out (right panel). Cortical suppression ratio is shown for ongoing activity (black trace) and during auditory and visual stimulation (red trace). Shades denote SD across mice (n = 15) (B) Differences in duration and variability of CSR change for wash-in and wash-out. Length of the bars denote duration of CSR change between 0.45 and 0.6 during wash-in (left panel) and wash-out (right panel) for individual mice (subjects 1 to 15). (C) Definition of five suppression levels by subdivision of the suppression ratio values into five categories. Suppression level is represented by a scale from 1 to 5, representing the increasing suppression of cortical activity between light anesthesia (level1) and deep anesthesia (level 5).

### Analysis of Sensory Evoked Activity

Trials were assigned to one of the five previously defined suppression levels. For each stimulation trial, the CSR value was determined. This was performed by calculating the CSR for the 30s interval prior to the time of stimulus onset. By this procedure a specific CSR value could be assigned to each trial. Then trials were grouped into one of the five suppression levels accordingly. LFP was rectified, pooled into positions in V1 and subiculum, and averaged for each mouse. For each animal, we calculated the evoked LFP potentials for each channel by averaging over all trials belonging to the respective anesthesia level 1–5, with the same CSR. Then we squared the resulting evoked potentials and averaged over cortical channels (2–7) and subiculum channels (12–15), resulting in cortical potential average and a subiculum potential average for each animal. We then calculated the sample statistic, by averaging potentials over animals. The latency was determined for the squared LFP signal. Trials belonging to one anesthesia level were averaged. This resulted in one averaged evoked LFP potential at each electrode position for each anesthesia level per animal. Latency was determined as the delay between stimulus onset and the half maximum between first peak and baseline. The number of animals in which valid responses could be recorded in V1 and subiculum differed for different anesthesia levels, as responses were not always evoked by the respective stimulus. For visual stimulation in V1 numbers for anesthesia level 1–5 were: n = 14, 15, 15, 15, 15. For auditory stimulation in V1 numbers for anesthesia level 1–5 were: n = 0, 0, 0, 8, 10. For visual stimulation in the subiculum numbers for anesthesia level 1–5 were: n = 0, 0, 3, 11, 10. For auditory stimulation in the subiculum numbers for anesthesia level 1–5 were: n = 0, 0, 6, 11, 10. Multiunit response rate was determined for the one second stimulus presentation interval by counting occurrence of spikes in the respective interval. For each CSR level, trials were pooled and averaged. For color-coded depth profile plots, multiunit activity was binned into 10ms bins, and the mean baseline rate druing 1s before stimulus onset was subtracted. To test for significant responses multiunit rate was compared to the prestimulus baseline interval of one second with a two-sample two-tailed t-test with significance level of 5%.

**Table 1:** Cortical suppression ratio during different phases of wash-in/wash-out of isoflurane.

	Start of wash-in	End of wash-in	Start of wash-out	End of wash-out
Condition				
ongoing	0.36 (0.03)	0.84 (0.06)	0.84 (0.05)	0.36 (0.04)
stimulation	0.33 (0.05)	0.79 (0.07)	0.79 (0.06)	0.37 (0.05)

Mean CSR (values ± SD) at the start and end of wash-in and wash-out for ongoing and sensory stimulation condition for all mice (n = 15). Between the end of the wash-in phase and the start of the wash-out phase the recording was interrupted for a short time (<30s), therefore the values are listed separately.

### Frequency Analysis

Time-frequency spectra of LFP signals were computed using multi-taper estimation in Matlab with the Chronux package (http://chronux.org/, [21]). For time-frequency spectra, we used a window size of five seconds with a step size of four seconds. To determine the peak frequencies during light anesthesia (CSR <0.5), power spectra for determining peak frequencies of LFP signals were computed using Welch’s averaged modified periodogram with a window size of 1s and a window overlap of 0.95s. To determine the repetition rate of burst events during burst-suppression, time-frequency spectra for frequencies <0.5 Hz were calculated in two steps. First, the envelope of the LFP signal was determined as the magnitude of the analytical signal, derived with a Hilbert transform. Then the time-frequency spectrum was determined from the envelope signal with window size of 60 seconds and a step size of one second.

**Figure 4:**
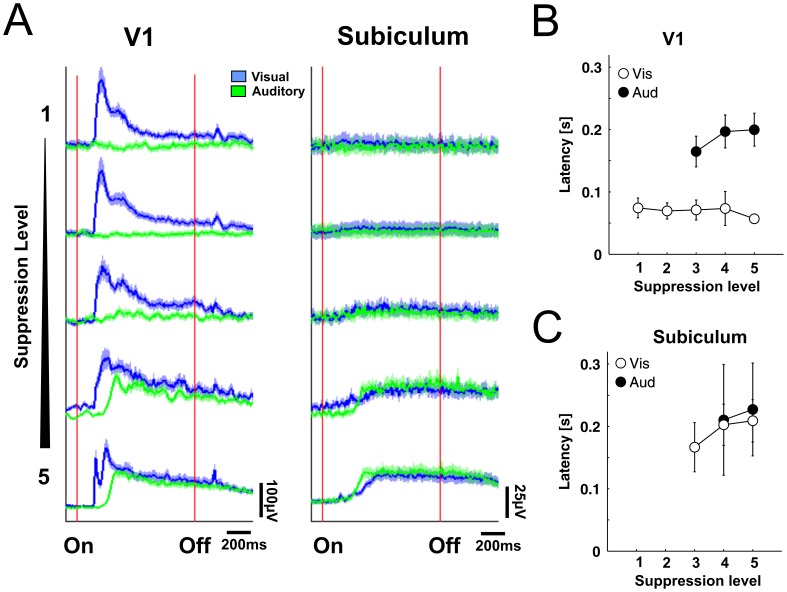
Auditory evoked LFP responses in V1 during deep anesthesia. (A) Evoked LFP responses in V1 (left panel) and subiculum (right panel) during increasing levels of anesthesia. Responses for visual stimulation (blue) and auditory stimulation (green) are overlayed. Red lines mark stimulus onset and offset. Colored shades denote SEM across mice (n = 15). (B) Onset latencies of LFP responses for visual (white circles) and auditory stimulation (black circles) in V1 (top) and subiculum (bottom) for increasing anesthesia levels. Errorbars denote SD across mice. For each level a different number of animals entered the calculation of mean latencies (see methods).

### Correlation Analysis

We determined the signal cross-correlation between all possible electrode site pairs from one probe. The correlation coefficient for the cross-correlation between the respective electrode signals was calculated by computing the normalized covariance function at zero lag, utilizing the MATLAB function xcov. Cross-correlation between electrode pairs was computed for non-overlapping 60s segments of the rectified filtered continuous MUA signals of the wash-in/wash-out cycle. Mean correlation was then determined by averaging all signal pair correlations between sites in V1, between sites in the subiculum, as well as for all signal pair cross-correlations between sites in the subiculum and V1.

**Table 2:** Onset latencies [ms] of sensory evoked activity during different anesthesia levels.

		Anesthesia Level				
		Level 1	Level 2	Level 3	Level 4	Level 5
V1	Visualstimulation	74 (16)	69 (13)	70 (16)	73 (27)	56 (5)
	Auditorystimulation	–	–	164 (24)	196 (26)	199 (16)
Subiculum	Visualstimulation	–	–	–	210 (80)	227 (74)
	Auditorystimulation	–	–	166 (39)	202 (33)	209 (33)

Comparison of onset latencies [ms] in V1 and the subiculum for visual and auditory responses at all anesthesia levels (mean±SD) for all mice (n = 15). Blank fields designate that no sensory evoked responses were present at the respective anesthesia level.

## Results

We recorded the depth profile of local field potentials (LFP) and extracellular multiunit activity in the mouse primary visual cortex and the subiculum using 16 channel linear probes (Figure 1A). We continuously recorded the change in neuronal activity from light to deep anesthesia after increasing isoflurane concentration from 0.7% to 2.5% (wash-in phase) until long periods (>30s) of cortical suppression appeared in the LFP (Figure 1B). Then, we recorded the return of neuronal activity to light anesthesia after decreasing isoflurane concentration back to 0.7% (wash-out phase). In each animal, this wash-in/wash-out cycle was repeated twice to measure ongoing activity and responses to visual and auditory stimulation.

**Figure 5:**
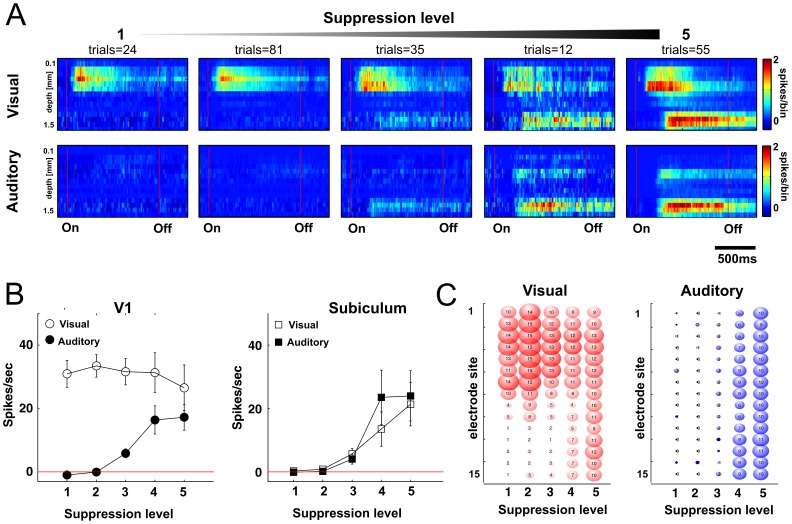
Anesthesia level alters response properties in V1 and the subiculum. (A) Depth profiles of multiunit responses in one mouse. Color-coded plots show averaged responses in each of the five anesthesia levels (from left to right). Note that baseline activity was subtracted from the response, thus the prestimulus interval shows no spontaneous activity. Flash stimulus (upper row) and tone stimulus (lower row) lasted one second. Numbers above color plots represent the number of trials in each group. (B) Absolute response rate change of multiunit activity to pre-stimulus baseline activity (N = 15, mean ± SEM) in V1 and subiculum after auditory and visual stimulation. (C) Significant increase of multiunit rate from baseline activity at respective electrode sites (paired t-test, p<0.05). The sizes of the circles correspond to the number of mice, where significant increase of responses occurred. Note that during deep anesthesia, sites at the border between visual cortex and the subiculum also show significant responses. Numbers of mice, where a significant increase of multiunit rate occurred are indicated inside the circles (total n = 15). Depth profile is shown for electrode sites (rows) at increasing anesthesia level (columns).

### Ongoing Activity

In all mice, LFP activity evolved in the same typical sequence during one wash-in/wash-out cycle (Figure 1C, 2A). The increase of isoflurane concentration to 2.5 vol % transformed the LFP activity typical for light anesthesia (Figure 2B, first panel) into increasingly synchronized slow rhythmic patterns with gradually increasing peak amplitudes (Figure 2B, second to fifth panel). After several minutes, activity in V1 and the subiculum first synchronized in a common rhythm characterized by short periods of electrical silence, alternating with high amplitude activity (Figure 2B, third panel). Then, silent periods in V1 progressively increased (cortical suppression), leading to an irregular pattern of burst-suppression. In contrast, activity in the subiculum did not express prolonged silent periods, but continued with a regular rhythm of burst-suppression (Figure 2B, fourth panel). The remaining irregularly appearing bursts in V1 synchronized with the regular subicular bursts, and never appeared during the subicular silent periods without a burst in the subiculum. However, subicular bursts appeared also in the absence of cortical bursts.

**Figure 6:**
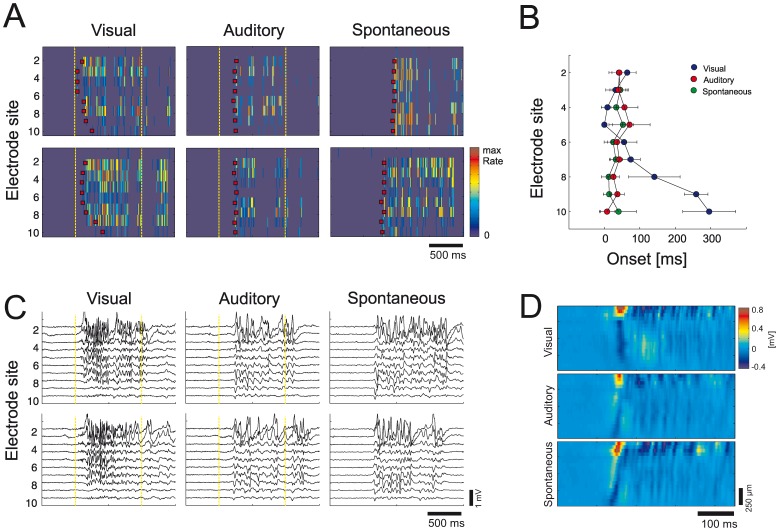
Depth profiles of auditory and spontaneous bursts are similar. (A) Multiunit depth profiles of individual visual, auditory and spontaneous bursts during deep anesthesia from recording in one mouse. Red squares denote onset of activity. Auditory and spontaneous bursts have similar depth profile, whereas they differ in comparison to visual evoked bursts. Images show color-coded histograms with 10ms bin size normalized to the maximum rate. Yellow dotted lines denote stimulus onset and offset. (B) Averaged multiunit depth profiles for activity onset. Individual depth profiles of each group were aligned to the earliest onset before averaging. Visual burst activity originates from the middle layers, whereas the onset of auditory and spontaneous burst activity is shifted towards lower layers of the visual cortex. Errorbars denote SD (n = 10). (C) LFP depth profiles of individual visual, auditory and spontaneous bursts during deep anesthesia from recording in one mouse. Yellow dotted lines denote stimulus onset and offset. (D) Averaged LFP depth profiles. Individual depth profiles of each group were aligned to the first positive peak of the upmost LFP signal (n = 10). Color-coded images were smoothed for better visibility.

**Figure 7:**
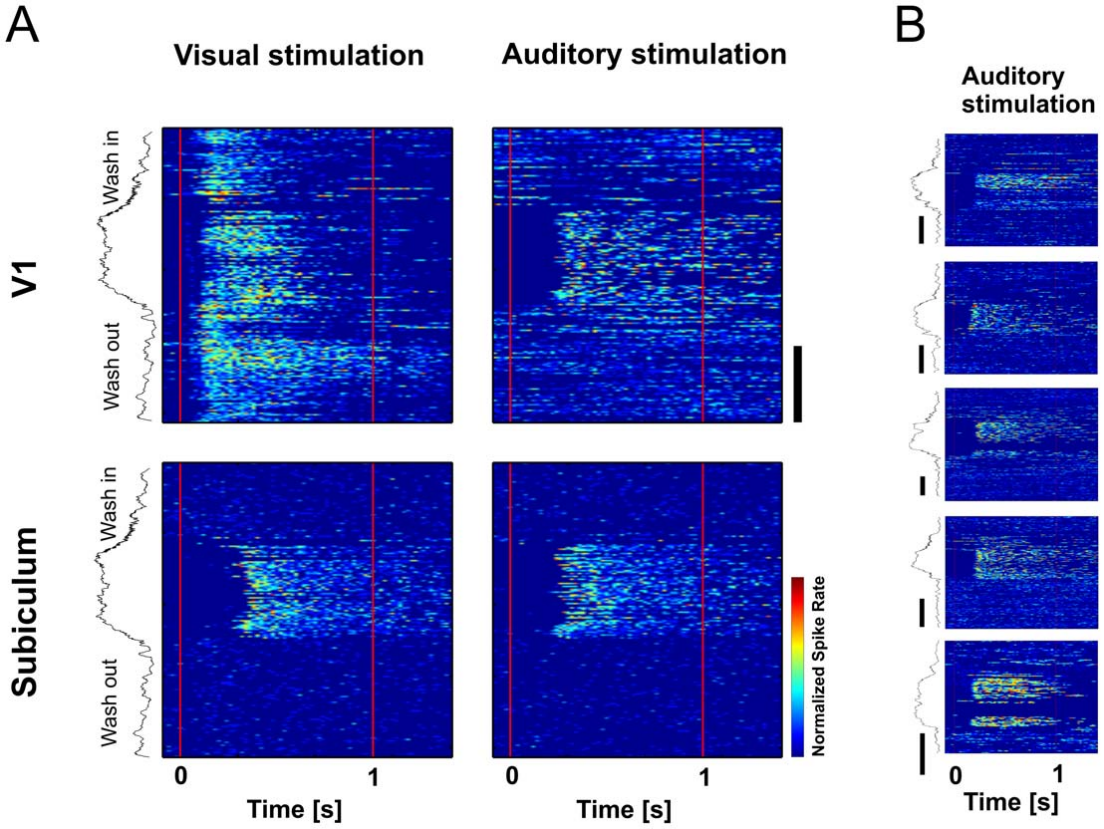
Discontinuous change in excitability in V1 and the subiculum between light and deep anesthesia. (A) Single trial responses of multi units to visual (left) and auditory (right) stimulation during one cycle of isoflurane wash-in and wash-out. Color-coded plots show responses in V1 (top row) and subiculum (bottom row). Red lines indicate stimulus onset and offset. Trials are ordered from top to bottom. Corresponding CSR (black trace) is plotted on the left side. Trials were separated by 10s interstimulus intervals. (B) Examples of auditory evoked responses in V1 during deep anesthesia from five different mice. Color coded plots show multi unit responses in V1 of one cycle of isoflurane wash-in and wash-out corresponding to right upper panel in (A). Time bars indicate 10min.

After the decrease of isoflurane concentration to 0.7 vol %, this sequence of events appeared in reversed order: suppression was followed by burst-suppression, then slow rhythmic activity to finally return into the initial state of small amplitude LFP activity. The return to light anesthesia consistently occurred around five minutes after decrease of isoflurane concentration. Multiunit activity in V1 and the subiculum followed these changes in LFP activity with analogous changes in spiking patterns. LFP bursts in V1 or the subiculum were always accompanied with bursts of strong multiunit activity at the respective electrode sites. This also shows that the described LFPs reflect localized activity of the respective regions (Figure 2C).

**Figure 8:**
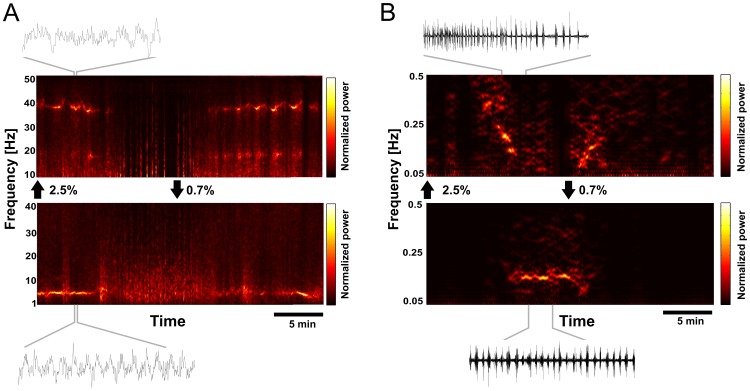
Time-frequency analysis of ongoing LFP activity. (A) Time-frequency plots of LFP activity in V1 (upper panel) and subiculum (lower panel) during wash-in/wash-out cycle. Black arrows between panels depict the onset of wash-in and wash-out. Note that frequencies are shown only from 10–50 Hz for V1, and frequencies from 1–40 Hz for the subiculum. Examples of raw signals (3s interval) are shown above and below the panels. (B) Time-frequency plots for V1 (upper panel) and subiculum (lower panel) for frequency range below 0.5 Hz. Note that different to (A) in (B) the time-frequency analysis was applied to the envelope of the LFP signal in order to analyze the presence and frequency of recurring burst events (see methods). Examples of raw signal (140s interval) are shown above and below the panels.

### Assessment of Anesthesia Depth with Cortical Suppression Ratio

To quantify anesthesia depth during the wash-in/wash-out cycle, we introduced a cortical suppression ratio (CSR), a measure based on the burst suppression ratio to quantify the proportion of suppression in cortical LFP activity (see methods, [22]). The CSR consistently captured the effect of isoflurane concentration on ongoing cortical LFP activity and changed reproducibly in all mice (n = 15, Figure 3A). CSR increased monotonically from a mean of 0.36 (SD 0.02) during low isoflurane concentration at the beginning of the recording towards a mean of 0.84 (SD 0.06) at the end of the wash-in phase. CSR then consistently returned to a mean of 0.35 (SD 0.05) after the wash-out phase. Sensory stimulation did not significantly affect the level of the CSR (Figure 3A, red trace, Wilcoxon rank sum test, p>0.05, Table 1). The time course of changes in the CSR was different between wash-in and wash-out. In both conditions, activity changes occurred faster and more uniform during wash-out than during wash-in (Fig. 3A). In order to quantify these differences in the time course, we determined the duration for the change in CSR during wash-in to raise from 0.45 to above 0.6, and compared it to the duration for the CSR to fall from 0.6 below 0.45 during wash-out (Figure 3B). Duration of CSR change between 0.45 and 0.6 was significantly longer during wash-in with 174s (SD 100) than the mean duration of 60s (SD 36) during wash-out (Wilcoxon rank sum test, p<0.01).

**Figure 9:**
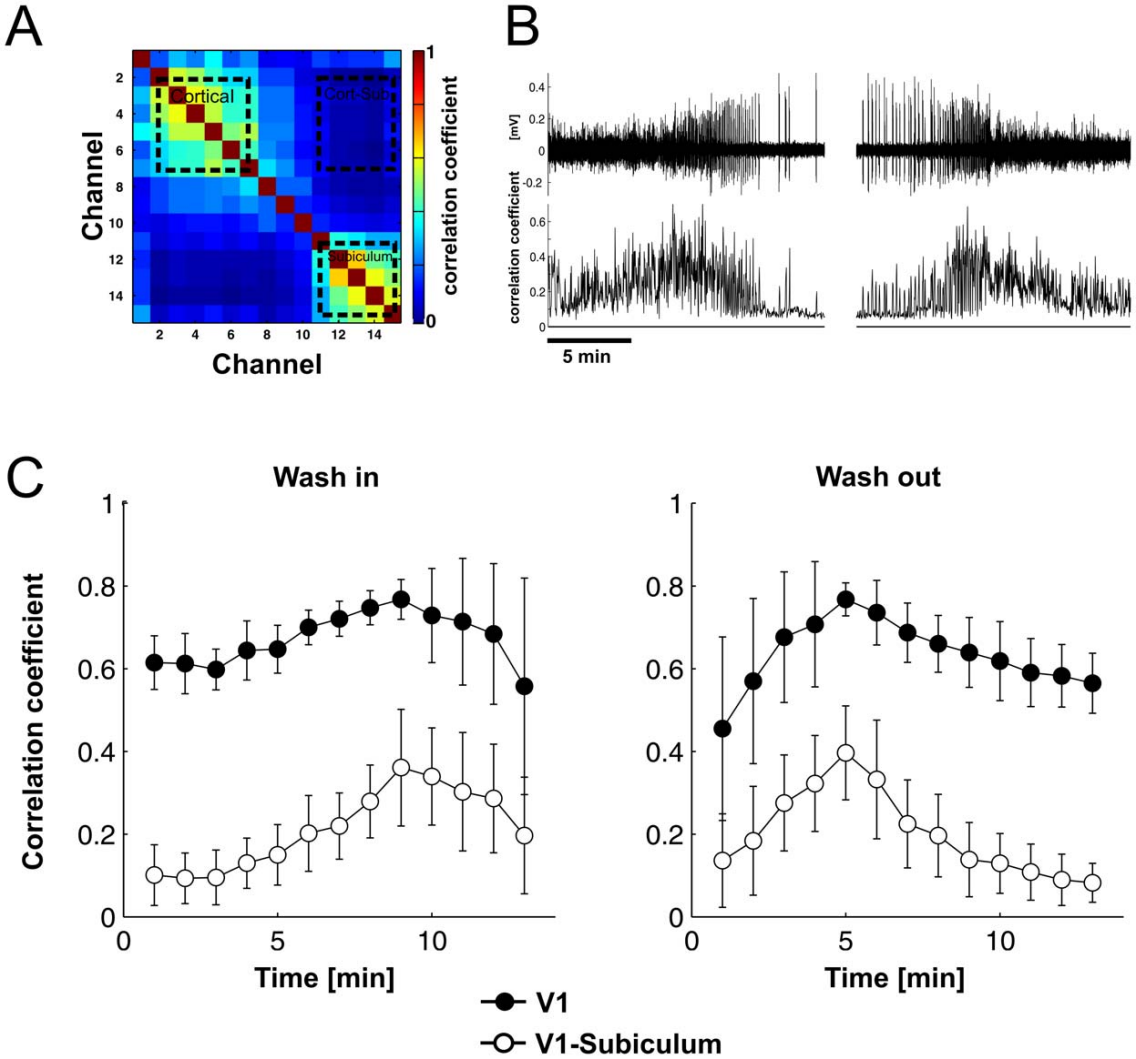
Intracortical cross-correlation changes with anesthesia level. (A) Cross-correlation matrix for MUA channels were calculated for 30s windows of the ongoing activity. The cross-correlation matrix is symmetric around the diagonal. Here, the example is shown for the cross-correlation between sites in visual cortex during light anesthesia at the beginning of the wash-in phase in a single mouse. V1 and the subiculum can be differentiated due to high correlation within, but low correlation between both structures (dotted rectangles). Mean cross-correlation was calculated for sites in V1, subiculum and V1-subiculum. (B) Example of ongoing LFP in V1 (top) with development of mean cross-correlation of sites in V1 over time with sliding windows with a window size of 30 s and a step size of 1s. (C) Mean cross-correlations for all animals for V1, subiculum and between V1 and subiculum during wash-in and wash-out. Errorbars denote SD across mice (n = 15).

### Auditory Responses in Visual Cortex during Deep Isoflurane Anesthesia

In a second condition, alternating visual and auditory stimuli were presented every 5 seconds continuously during a full wash-in/wash-out cycle of isoflurane. To analyze the effect of the visual and auditory stimulation, we classified and pooled responses into one of five suppression levels with respect to their CSR values between light anesthesia/low suppression level (CSR <0.4) and deep anesthesia/high suppression level (CSR >0.7, Figure 3C). That allowed to average the stimulation trials obtained in each suppression level, and thus to compare the effect of increasing isoflurane concentration on visual and auditory evoked responses in V1 and the subiculum (Figure 4A). The suppression level increases and decreases with anesthetic isoflurane concentration and thus generally corresponds to anesthesia depth, with a low suppression level during light isoflurane anesthesia and a high suppression level during deep isoflurane anesthesia.

In V1, visual stimulation evoked responses during all suppression levels. The response was characterized by a short latency response during light anesthesia, which changed into a two-component response with a short latency and a long latency component during deep anesthesia with high suppression level. The mean latency of the first component was significantly reduced from 74ms (SD 16ms) during light anesthesia to 56ms (SD 5ms) during deep anesthesia (direct comparison between Level 1 and 5, Wilcoxon rank sum test, p<0.01, Figure 4B, Table 2). The second long latency component observed during deep anesthesia had a mean latency of 130ms (SD 16ms).

Auditory stimulation had no measurable effects in V1 during light anesthesia, but elicited long latency responses during deep anesthesia at suppression levels above CSR >0.5 (Figure 4A, level 3–5). The mean latencies of these auditory evoked responses increased from 164ms (SD 24ms) at level three to 199ms (SD 16ms) at level five. They were significantly longer than visual response latencies during the respective suppression level (Figure 4B, Table 2, Wilcoxon rank sum test, pairwise comparison, p<0.01). At the same time as auditory responses occurred in V1, both visual and auditory stimulation evoked responses in the subiculum with similar latencies (Figure 4A). The latency differences were not significant between stimulation modalities with a mean of 227ms (SD 74ms) for visual and 209ms (SD 33ms) for auditory stimulation during the highest suppression level during deep anesthesia (Figure 4C, Wilcoxon rank sum test, pairwise comparison, p>0.05, Table 2).

In addition to LFP responses, increasing anesthesia depth had a similar effect on multiunit responses in V1 and the subiculum. The changes in excitability were well reflected in the depth profile of multiunit activity (Figure 5A). Visual responses in V1 were present during all anesthesia levels, whereas auditory stimulation first slightly suppressed activity in V1 during light anesthesia, and then elicited auditory multiunit activity with increase of anesthesia depth at the same suppression levels as LFP bursts appeared (CSR>0.5, Figure 5B, left panel). At the same time, bursts could be evoked in the subiculum by visual and auditory stimulation (Figure 5B, right panel).

Visual multiunit responses were reliably present at electrode positions located in V1 during light anesthesia in all animals (CSR<0.5, Figure 5C, difference to pre-stimulus baseline activity, two-sided t-test, p<0.05). During deep anesthesia, the reliability of neuronal responses to sensory stimulation decreased and visual or auditory stimulation did not evoke significant activity in all of the animals. However, if visual or auditory responses were present, they evoked activity in all electrodes over the whole shank in an unspecific way, that did not reflect the border between V1 and the subciulum (Figure 5C). Thus, deep layer VI neurons and subicular neurons at the border to white matter seem to become active during synchronized burst events during deep anesthesia, which are inactive during light anesthesia.

We also compared individual depth profiles of multiunit activity of visual, auditory and spontaneous bursts in visual cortex (Figure 6A, B). Multiunit depth profiles of auditory and spontaneous bursts were generally similar, with earliest activity originating in lower cortical layers. In contrast, earliest onsets of visual evoked bursts were originating from upper and medium layers. The similarity of auditory and spontaneous bursts was also reflected in LFP depth profiles (Figure 6C, D).

### Discrete Change of Response Properties between Trials

To identify the time point of the transition in excitability, we analyzed single trial responses during the wash-in/wash-out cycle. The responsiveness to sensory stimuli between light and deep anesthesia did not change gradually. Rather, the change in response properties appeared within two consecutive trials during wash-in, and disappeared with a similar discontinuity during wash-out (Figure 7A). The change towards auditory excitability in V1 happened in parallel to the change in excitability of the subiculum. This state change could be easily identified in most of the recordings and occurred consistently after CSR increased above 0.5 (mean: 0.58, SD: 0.04), and then disappeared when CSR dropped below 0.5 (mean: 0.53, SD: 0.04). A CSR >0.5 generally indicated the presence of distinct burst-suppression. Sensory stimuli sometimes ceased to elicit activity during prolonged high CSR levels, but auditory evoked burst responses often reappeared during wash-out, shortly preceding the return to the light state (Figure 7B). The return was marked by a sudden strong increase in ongoing multiunit activity most obvious acoustically on the audio monitor of the spike signals. From this point on, only visual evoked responses remained in visual cortex.

### Dichotomy of Rhythms between Light and Deep Anesthesia

The two response states could be characterized by the patterns of ongoing activity. Time-frequency analysis revealed a dichotomy in dominant frequencies and recurring patterns of the ongoing LFP (Figure 8). The light anesthesia state was characterized by theta oscillations in the subiculum with a mean peak frequency of 4.8 Hz (SD 0.3 Hz) (Figure 3A, lower panel). In V1, the light state was marked by transient periods of gamma oscillations with mean peak frequency of 33.9 Hz (SD 2.1 Hz) (Figure 8A, upper panel) and by delta-oscillations with mean peak frequency of 2 Hz (SD 0.4 Hz) (low frequencies are not shown in Figure 8A for better visualization of ongoing gamma oscillations in the color-coded plot). To analyze the frequency of the recurring events of the bursts, we similarly applied time-frequency analysis to the envelope of the LFP signal during the wash-in/wash-out cycle (Figure 8B). The deep anesthesia state was characterized by regular slow rhythmic burst-suppression in the subiculum, with a mean peak frequency of 0.17 Hz (SD 0.05 Hz) (Figure 8B, lower panel). V1 activity did not reflect this rhythm, but exhibited the above mentioned, progressive lengthening of silent periods during increase of isoflurane, and a shortening of silent periods during decreasing levels of isoflurane. This concentration-dependent change of interval length was reflected in a frequency-modulated band in the time-frequency plot of LFPs in V1 (Fig. 8B, upper panel). The continuous wash-in/wash-out cycle between high (2.5%) and low (0.7%) isoflurane concentration thus produced two general states with characteristic frequencies occurring on a macroscopic time scale. They could be distinguished by a slow rhythm in the deep state and fast rhythms in the light state.

### Build Up in Cortical Correlation Preceding Transition between States

We additionally computed correlations between electrode signals and determined the mean correlation for signals within V1, within subiculum and between V1 and subiculum (Figure 9A, B). Increase of isoflurane concentration induced a gradual increase of signal correlation in V1. Its maximum indicated the beginning of the burst-suppression phase (Figure 9C). Subiculo-cortical correlation increased similarly, also with a maximum at the onset of burst-suppression. The mean correlation decreased after the onset of burst-suppression. This happened because the signals during the silent periods of suppression mainly reflect uncorrelated background noise. Although signal correlation is high during the bursts, the combination with the interposed suppression periods yields a low mean correlation value. With increasing suppression periods the correlation then decreases accordingly. This behavior was reversed during wash-out, when correlation again first reached a maximum and then decreased towards its lowest values during light anesthesia at the end of the wash-out. In consequence, the peak in the correlation indicates the state transition between light and deep anesthesia, preceded by a gradual change of correlations during the light state.

## Discussion

We demonstrated that auditory evoked burst activity in V1 could be elicited after a state transition during deepening of isoflurane anesthesia. This heteromodal auditory-evoked activity in V1 had latencies of ∼200ms. At the same anesthetic depth both auditory as well as visual evoked responses appeared in the subiculum. This anesthetic state was characterized by a regular rhythm of burst-suppression (∼0.2 Hz) in the subiculum and a less regular burst-suppression pattern in V1.

### Visual Responses in V1 during all Anesthesia Levels

During all anesthesia levels visual responses could be observed in V1, with onset latencies in the range previously described in anesthetized mice [23]–[25]. Interestingly, the shortest latencies occurred during deep anesthesia (Table 2). This indicates that deep anesthesia rather speeds up than slows down visual transmission to V1 (Figure 4). The initial short latency visual evoked response was continued by a long lasting second component during deep anesthesia, which was not coupled to stimulus offset. This second component resembled spontaneous bursts during burst-suppression. Sensory evoked cortical bursts during burst-suppression have been observed previously [11]–[16]. Our results indicate that visual evoked bursts in V1 during burst-suppression are likely to be directly triggered by a short latency visual response.

### Origin of Auditory Evoked Activity in V1

If burst events in V1 are triggered by visual stimulation during deep anesthesia, then auditory evoked bursts are likely to occur in A1 in a similar fashion. Auditory evoked burst responses in V1 possibly originate from cortical spreading activity from A1 into V1. Spreading activity in neocortex of anesthetized rodents in vivo and in slices of ferret neocortex exhibit speeds in the range of 10–100 µm/ms [26]–[29]. Propagating activity within this speed range, crossing the distance from A1 to V1 (∼4mm estimated) would then produce the observed activity in V1 after ∼200ms (Table 2). Cross-modal propagating waves [30], as well as propagating waves between visual cortical areas [30], [31] have been described in a similar speed range in rats. Importantly, both studies explicitly used high isoflurane concentration levels (>1.5%). This suggests that such sensory evoked propagating waves occur only during deep anesthesia, and during this state, activity propagates freely across and beyond the typical functional areal borders. The similarity of the depth profiles of auditory evoked bursts and spontaneous burst activity further supports this view (Figure 6).

The long latency auditory burst response in the subiculum (∼200ms) indicates that the sensory evoked activity during deep anesthesia also propagates from primary sensory cortex beyond the cerebral cortex surface. The subiculum, serves as a mediator of information between the hippocampus and cortical regions [32]–[34]. Subicular activity is delayed with respect to neocortical activity during urethane anesthesia in rats [35], and a lag of ∼180ms between cortical and hippocampal activity has been described in urethane anesthetized mice [36]. During prefrontal cortical bursts in anesthetized rats, information flow is directed from the cortex towards the hippocampus [37].

An alternative mechanism of the auditory burst response in V1 during deep anesthesia could be the activation via sparse direct projections from auditory cortex [38], or via an indirect pathway from primary auditory cortex into V1 [39]. However, the observed latency of more than 150ms for auditory responses in V1 exceeds the latency that would be predicted from typical conduction velocities for cortico-cortical axons [40]. Also it remains unclear why these projections would be effective only during deep anesthesia. Both of these objections similarly apply for the subicular responses. Initiation of sensory evoked bursts by unspecific ascending thalamic or midbrain projections, as another possibility do not explain the presence of the short latency visual response in V1, and the latency differences between visual and auditory evoked bursts in V1 (Figure 4 and 5, Table 2).

### Relationship between Burst-Suppression and Slow Oscillation

Although bursts can be evoked by sensory stimulation during burst-suppression [11]–[16], burst-suppression is also spontaneously present in cortical slices [41] and in isolated cortex in vivo [42], [43]. Burst-suppression can also be induced by high concentrations of all common anesthetics, such as isoflurane, halothane, sevoflurane, propofol, etomidate, thiopental, ketamine and pentobarbital [9], [44]. In anesthesia-induced burst-suppression, the duration of silent periods between bursts is dose-dependent [9], [45]–[47]. We also observed such a dose-dependent increase of suppression intervals towards extensive suppression of activity in V1 during wash-in (Figure 2A). Interestingly, the suppression intervals in the subiculum did not show this dose-dependency, and bursts in the subiculum mostly occurred at a regular frequency of ∼0.2 Hz independent of anesthesia level (Fig. 2B) even during suppression of cortical activity. During this anesthesia level, the irregular spontaneous bursts in V1 were always linked to the rhythmic bursts in the subiculum (Figure 2B and 8B). Uncoupling between cortical and hippocampal burst events have been described also during thiopental anesthesia in rats [47].

Slow frequency activity in hippocampus of urethane anesthetized rats [48], and etomidate anesthetized mice [49] has been observed at ∼1 Hz. The slow burst-suppression rhythm of ∼0.2 Hz we observed in the subiculum is considerably slower and falls in the frequency range of cortical slow oscillations. Slow oscillations in neocortical activity exhibits frequencies of ∼0.5 Hz in urethane anesthetized mice [36], ∼0.3 Hz in halothane anesthetized cats [27], 0.3–0.4 Hz for urethane anesthesia and 0.6–1 Hz during ketamine anesthesia [50]. A dose-dependent decrease in frequencies between 0.5–0.15 Hz have been observed in cortex of isoflurane anesthetized rats [51]. Unfortunately, the distinction between slow oscillations and burst-suppression is not always delineated in the literature, it remains to be shown, whether these two phenomena share a common neuronal background.

### State Transition Hypothesis

We found that cortical responses did not change gradually during increase of isoflurane concentration, but the transition in response properties was discontinuous. Response properties changed between two trials, corresponding to an interval of 10s (Figure 9A), and the same fast change occurred during return to light anesthesia. In the ongoing condition, this return was often demarcated by a sudden increase of cortical activity, also reflected by a sharp edge in the CSR curve (Figure 3A and 7). The forward and return path between anesthesia levels differed. Whereas the return path to light anesthesia was highly uniform, the forward path into deep anesthesia was slower and more variable (Figure 3B). This behavior mirrors the difference in forward and reverse paths of anesthesia induction and emergence, recently termed ‘neural inertia’ [52].

In the late stages of the wash-in phase, sometimes only the short-latency visual response in V1 remained without eliciting a sensory evoked burst. The atypical changes in cortical network excitability thus only occur in a critical window of isoflurane level, and are induced in some way by the effects of isoflurane on properties of its target neurons, an enhancement of GABA responses [53]–[55], and inhibition of NMDA receptor function [56], [57].

Below the critical isoflurane level, the cortical network returns to a stable state, reflected by the prominent ongoing theta oscillations in the subiculum and gamma oscillations in V1 (Figure 8). Above the critical level, isoflurane produces a stable slow rhythm (∼0.2 Hz) in the subiculum (Figure 8B), and it is clear that the change in excitability is inherently linked to the presence of burst-suppression, which was predicted by a CSR>0.5. Although we observed a rather mutually exclusive dichotomy between these two states, it is important to note that this distinction becomes distinct on a long time scale of our observation window. Whether the state change can be demonstrated on a faster time scale remains to be studied.

However, we showed that a gradual change in isoflurane concentration eventually leads to a discontinuous global change in cortical network properties. From this point, neural subsystems seem to be ‘short-circuited’ and ‘permeable’ to freely propagating waves of sensory evoked activity that are able to produce cross-modal responses. This is clearly an effect of anesthesia level, and therefore has methodological implications for in-vivo recordings carried out under anesthesia. An increased focus on monitoring anesthesia depth might prevent unnoticed crossings between network states and might explain certain aspects of variability in neurophysiological data.
